# Usefulness of Traditional Serum Biomarkers for Management of Breast Cancer Patients

**DOI:** 10.1155/2013/685641

**Published:** 2013-11-07

**Authors:** Peppino Mirabelli, Mariarosaria Incoronato

**Affiliations:** Fondazione SDN IRCCS, Via E. Gianturco 113, 80143 Naples, Italy

## Abstract

The measurement of serum tumor markers levels in breast cancer (BC) patients is an economic and noninvasive diagnostic assay frequently requested by clinical oncologists to get information about the presence or absence of disease as well as its evolution. Despite their wide use in clinical practice, there is still an intense debate between scientific organizations about the real usefulness for patient monitoring during followup as well as response to therapy evaluation in case of advanced BC. In this review, we want to highlight the current recommendations published by scientific organizations about the use of “established” BC serum markers (CEA, TPA, TPS, CIFRA-21, CA15-3, and s-HER2) in clinical oncology practice. Moreover, we will focus on recent papers evidencing the usefulness of tumor markers levels measurement as a guide for the prescription and diagnostic integration of molecular imaging exams such as those performed by hybrid 18-fluorofeoxyglucose-positron emission tomography with integrated computed tomography. This technology is nowadays able to detect early cancer lesions undetectable by conventional morphological imaging investigation and most likely responsible for increasing of serum tumor markers levels.

## 1. Introduction

Serum tumor markers are soluble molecules released into the blood stream by cancer cells or other cell types belonging to tumor microenvironment [1]. The measurement of these molecules is considered an economic and noninvasive diagnostic assay able to give information about the presence or absence of disease as well as its evolution. In particular, the ideal serum tumor marker should be able to (i) early detect disease; (ii) predict response or resistance to specific therapies; (iii) monitor the patient after primary therapy [2]. In case of breast cancer (BC), different serum markers were tested for these purposes, and to date, the most used in clinical practice are carcinoembryonic antigen (CEA), the soluble form of MUC-1 protein (CA15-3), circulating cytokeratins such as tissue polypeptide antigen (TPA), tissue polypeptide specific antigen (TPS) and cytokeratin 19 fragment (CIFRA-21-1), and the proteolytically cleaved ectodomain of the human epidermal growth factor receptor 2 (s-HER2). Although all of these markers are routinely used in clinical practice, none is useful for screening programs and/or early diagnosis of BC [1, 2]. In addition, an intense debate is still present between scientific organizations regarding their usefulness for patient monitoring during follow-up as well as evaluating response to therapy in case of advanced BC. Nevertheless, thanks to the introduction in clinical practice of molecular imaging exams able to identify cancer lesions previously undetectable by conventional morphological imaging instruments, tumor markers are now reevaluated as an early warning able to highlight patients at risk to relapse [3]. The aim of this review is to highlight the current recommendations about the use of “established” serum markers (CEA, TPA, TPS, CA15-3, and s-HER2) as well as to discuss their usefulness for the prescription and diagnostic integration [4] of molecular imaging exams such as those performed by hybrid 18-fluorodeoxyglucose-positron emission tomography with integrated computed tomography (FDG-PETCT).

## 2. Established Biomarkers: Structure and Function

### 2.1. Carcinoembryonic Antigen

**Figure 1:**
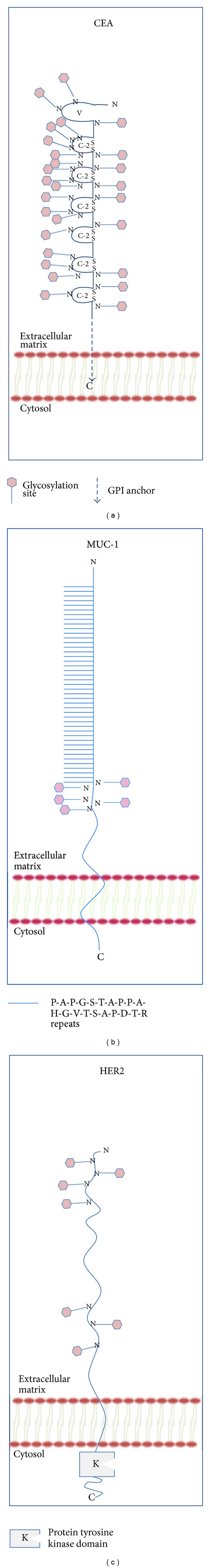
Schematic representation of CEA (a), MUC-1 (b), and HER2 (c) proteins. Of note, proteins are not to scale.

In a historical paper published in 1965, Gold and Freedman identified an antigen absent in human normal adult colon specimens and brightly displayed in human fetal and cancer colon tissues; therefore, they called this antigen carcinoembryonic antigen (CEA) [5]. About 30 years later, it was found that CEA consists of a large family of glycoproteins whose structure was similar to that of immunoglobulin super family [6]. Nowadays, CEA antigen is known as cluster of differentiation (CD)66e or CEACAM5 [6, 7]. This protein, with a size of about 100–200 kDa, is a member of the immunoglobulin superfamily with an N-terminal domain including 29 potential glycosylation sites and is attached to the membrane by a glycosyl phosphatidylinositol (GPI) anchor [6, 7]. As reported in Figure 1(a), the extracellular region is composed of six domains homologous to the immunoglobulin constant domain of the C-2 set (IgC2-like) and one immunoglobulin variable domain (IgV-like) [6, 7]. The mechanism responsible for its release in the extracellular matrix is still object of study; however, *in vitro* experiments disclosed that CEA, like other GPI anchored proteins, could be released due to the GPI anchor cleavage catalysis mediated by an endogenous glycosylphosphatidylinositol-specific phospholipase D (GPI-PLD) type enzyme [8].

The function of CEA is still not completely understood. Most probably, it is involved in adhesion to the extracellular matrix and to other cell types thanks to the homophilic and heterophilic interactions with CD66a (CEACAM1) and CD66c (CEACAM6) [9]. Interestingly, recent findings suggest its involvement also in cancer growth, invasion, and metastasis [10, 11]. Indeed, overexpression of CEA and CEACAM6 inhibits anoikis and apoptosis in colon and pancreatic cancer cells [12], disrupts cell polarization and tissue architecture [13], enhances liver metastasis [13], increases chemoresistance [14] as well as recombinant overexpression of CEACAM5 and -6 proteins in transgenic mice (CEABAC mice), and promotes the formation of colon tumours and lung tumours [15].

### 2.2. MUC-1 Protein

CA15-3 is the soluble form of MUC-1 protein, that is, a large type I transmembrane glycoprotein. As reported in Figure 1(b), MUC-1 is featured by a large tandem repeat domain highly polymorphic that can include a minimum of 21 up to 125 repeats between individuals; each repeat is composed of 20 amino acids rich in serine, threonine, and proline residues, and the cytoplasmic portion is composed of 72 amino acids containing 7 tyrosine residues forming a potential clathrin-mediated endocytic signal sequence [16]. The cytoplasmic tail of MUC-1 is involved in signal transduction by interaction with signaling molecules such as beta-catenin and growth factor receptor-bound protein/Son of Sevenless (Grb/SOS) [16]. Interestingly, MUC-1 is able to exceed the distance spanned by most cell surface proteins being this protein formed by a rigid structure that protrudes 200–500 nm from the cell surface [16].

As regards the functional role of MUC-1, initially, it was supposed to be mainly involved in the protection, lubrication, and hydration of external surfaces of epithelial tissue layers, as well as lining ducts and lumens in different parts of the body [16, 17]. Indeed, MUC-1 is strongly expressed by epithelia of glands and ducts as well as goblet and columnar cells of epithelial tissues where it has a protective role by inhibiting the microbial access to the cell wall and blocking degradative enzymes activity [17, 18]. Also, in case of cellular transformation, a growing number of pieces of scientific evidence proved that MUC-1 should be also considered *de facto* an oncogene. Indeed, its levels are upregulated in epithelial cancer cells of different origin and increase with cancer development and metastasis [18]. In particular, MUC-1, like other transmembrane mucins, contributes to oncogenesis by promoting receptor tyrosine kinase signalling, loss of epithelial cell polarity, constitutive activation of growth and survival pathways (e.g., the Wnt-*β*-catenin and nuclear factor-*κ*B pathways), and downregulation of stress-induced death pathways [19–22]. Moreover, it has a critical role for cancer immunosurveillance being able to block the access of immune cells to tumors, so that cancer cells are protected from possible clearance mediated by the immune system [23, 24]. Although MUC-1 expression is strictly associated with BC aggressiveness, it is not routinely performed for histological classification of BC, and its use in clinical setting is focused on the serum evaluation of its soluble form called CA15-3.

### 2.3. HER-2

The discovery of human epidermal growth factor receptor 2 (HER-2; also known as ERBB2) by King et al. in 1985 is considered a milestone for cancer research [25, 26]. Indeed, after its discovery, HER-2 gene was found to be amplified in different number of epithelial cancers, and its protein overexpression has been linked to central tumor cell proliferation and survival pathways. HER2 is a member of the ERBB tyrosine kinase receptor family that includes ERBB1 (EGFR), ERBB3 (HER3), and ERBB4 (HER4). The HER2 receptor is a type 1 transmembrane protein of 1233aa with an extracellular domain of 630aa containing seven potential N-linked glycosylation sites, a transmembrane region of 23aa, and a cytoplasmatic portion of 580aa with a tyrosine-kinase-containing domain (Figure 1(c)) [26].

Unlike the other members of ERBB family, no direct ligand binding has been observed for HER2 receptor, and it is known that its activation relies on (i) heterodimerization with another family member (i.e., EGFR upon EGF ligand binding) or (ii) homodimerization with itself when expressed at very high levels [27]. In case of heterodimerization, HER2 is necessary for ligand binding stabilization and phosphorylation of tyrosine residues that leads to downstream second messenger pathways activation such as those mediated by mitogen activated protein kinase (MAPK), phospholipase-C*γ* and phosphatidylinositol 3 kinase (PI3K) [26]. The homodimerization of HER2 is primarily detectable in case of cellular transformation that leads to HER2 overexpression, particularly in case of BC where HER2 gene was found amplified in 20% of cases up to 25–50 copies. This amplification is responsible for 40–100-fold increase in HER-2 protein resulting in 2 million receptors expressed at the surface of tumor cell [26]. The abnormal activation of HER2 in case of homodimerization in cancer tissues leads to a cascade of signaling events causing the activation of a series of transcription factors able to regulate many genes generally involved in cell proliferation, survival, differentiation, and invasion [26]. Due to these peculiar characteristics, the detection of HER-2 has become a routine prognostic and predictive factor in BC and is recommended by the American Society of Clinical Oncology/College of American Pathologists international guidelines [28].

### 2.4. Cytokeratins (TPA, TPS, and CYFRA 21.1)

Cytokeratins (CKs) are a class of intermediate filaments primary involved in cytoskeletal organization of epithelial cells for the fixation of the nucleus and maintenance of cellular morphology for cell protection from mechanical and nonmechanical stressors [29]. CKs comprise 20 related polypeptides classified in two groups: type I includes acidic CKs (CK 9–20) and type II includes neutral-basic CKs (CK 1–8) [29]. Type I and II CKs are always present in stoichiometric amounts, and their expression is differentiation dependent; for instance, in a lot of normal simple epithelial cells (glandular epithelia, transitional cell epithelium, and hepatocytes), CK8 and its obligate partner CK18 constitute the primary pair. The keratin expression pattern of normal epithelia is largely maintained also in the neoplastic counterpart. Therefore, keratins have long and extensively been used as immunohistochemical markers in diagnostic tumor pathology and most cancers of glandular epithelia origin, including BC, express CK8, CK18, and CK19 as specific cancer tissue biomarkers [29, 30]. Interestingly, during the last years, a growing number of pieces of experimental evidence disclosed that CKs have also an important role in cancer pathophysiology. In particular, in case of hormonally responsive BC, it has been shown that CK18 has a regulatory role as it can effectively associate with and sequester the estrogen receptor-alpha (ER-*α*) target gene and ER*α* coactivator LRP16 in the cytoplasm, thus attenuating ER*α*-mediated signaling and estrogen-stimulated cell cycle progression in BC cells [31]. Moreover, in case of BC, CK8 and CK18 are frequently found downregulated in metastatic tissue biopsies where their ubiquitin-immunoreactive degradation products are detectable and related with tumor aggressiveness [32]. Also, CK-8, -17, and -19 are upregulated in BC cells featured by defective autophagy, a condition where disease-promoting mechanisms such as toxic protein aggregation, oxidative stress, genomic damage, and inflammation are increased [33, 34]. In oncological patients, cytokeratins serum levels are informative of disease status and are frequently used for clinical management. For this purpose, the CKs tested primarily into the blood stream are CK8, CK18, and CK19 and the most widely used assays are (i) TPA for the evaluation of CK8, CK18, and CK19; (ii) TPS for the measurement of CK8 and CK18; and (iii) CYFRA 21.1 for CK19.

## 3. Tumor Markers for Diagnosis and Prognosis of BC

### 3.1. CEA

The first tumor marker used for diagnostic purposes of different human cancer (colorectal, pancreatic, breast, ovary, head and neck, bladder, kidney, and prostate cancers) was the CEA antigen, found overexpressed in serum of oncological patients compared to healthy individuals [35]. Further studies showed that CEA measurement was not useful for screening or for diagnosis of early BC since it was too insensitive and nonspecific to reliably differentiate patients with early BC from those with benign disease or disease free [36–38]. However, in case of symptomatic BC patients CEA sensitivity increases, and some authors evidenced that CEA levels at diagnosis are able to correlate with the stage of disease [39, 40]. Additionally, as a prognostic tool, the positive pretherapeutic levels of CEA may be useful to highlight those patients with a worse prognosis and at risk to have a recurrence after primary therapy [41, 42].

### 3.2. CA15-3

The soluble form of MUC-1 (CA15-3) was identified as a more specific BC marker with respect to CEA. Also, this marker disclosed low sensitivity and specificity for the detection of BC, since its sensitivity is 10–15%, 20–25%, and 30–35% for stages I, II, and III, respectively [43]. Therefore, the screening of CA15-3 in BC patients is not recommended. As for CEA, the increasing levels of CA15-3 may be useful to detect patients with advanced disease [44]. Indeed, the simultaneous positivity of both markers allows early diagnosis of metastases in up to 60–80% of patients with advanced disease [45].

### 3.3. s-HER-2

In the last ten years, particular attention has been devoted to the detection of the soluble form of HER-2 in serum from BC patients. Indeed, as demonstrated by several *in vitro* and *in vivo* experiments, the ectodomain of HER-2 can be proteolytically cleaved from the intact receptor and released as soluble molecule (s-HER-2) [46–48]. In normal healthy individuals, low concentrations of s-HER-2 can be detected in serum; however, in some BC patients, s-HER2 levels are increased according to the tumor burden and HER-2 status [49]. Even if s-HER-2, like other circulating tumor markers, has limited usefulness for diagnosis and/or screening of BC, the US Food and Drug Administration (FDA) introduced its serum levels measurement for monitoring trastuzumab treatment in BC patients with HER-2 positive tissue and serum expression [50]. Particularly, in these patients, it has been shown that decreasing values of s-HER-2 can be related to a positive response to biological therapy, whereas increasing levels are able to predict resistance or may act as an early warning indicating that standard doses of trastuzumab are insufficient [51].

### 3.4. Cytokeratins

The screening of circulating cytokeratins in BC patients at diagnosis is actually not recommended; however, recent observations showed that the detection rate of hepatic metastases in patients with BC can be raised up to 90% by simultaneous testing the serum levels of CA15-3, CEA, and circulating cytokeratins (TPA, TPS, and CYFRA 21.1) [45, 46].

## 4. Tumor Markers for Surveillance after Primary BC Treatment

### 4.1. International Guidelines Recommendations

Serum tumor markers are frequently required by clinical oncologists as an economic and noninvasive test for patient management during followup after primary BC therapy for an early detection of recurrence or metastases [63, 64]. They should be useful to discriminate those patients at risk to have a recurrence after primary BC treatment; however, their usefulness is still object of intense debate in the scientific community [63, 64]. This criticism has been raised during the 1990s, when two large multicenter randomized prospective trials, accounting each for about 1000 patients, showed that patients subjected only to periodic clinical visit and mammography showed the same outcome respect to those following an intense regimen including radiology and biomarkers screening [65, 66]. Furthermore, this *caveat* has been recently confirmed and stressed by the ASCO guidelines for Breast Cancer Follow-Up and Management After Primary Treatment (Table 1) [52]. In particular, these guidelines recommend that an optimal followup has to be primarily done by a careful history and physical patient examination performed by an experienced physician together with a regular mammography, particularly in case of breast conserving surgical therapy. Conversely, tumor markers exams, bone scans, chest radiographs, liver ultrasounds, CT, and even FDG-PET scanning as well as magnetic resonance imaging are not recommended by ASCO for routine BC followup in asymptomatic patients with no specific findings on clinical examination [52]. Despite these recommendations, other scientific organizations suggest serum tumor markers testing for postoperative surveillance as well as therapy monitoring in patients with advanced BC (Table 1). In particular, the European Group on Tumor Markers (EGTM; http://www.egtm.eu/recommendations.html) [57] and the National Academy of Clinical Biochemistry (NACB) [56] indicate that rising of tumor markers serum levels, with particular attention to CA15-3 in case of BC, is able to detect asymptomatic patients at risk to have metastases prior to the onset of clinical or radiological findings. In this way, the relationship between serum levels of biomarkers and imaging findings is still an argument of great interest for both laboratory medicine and radiology [3].

### 4.2. Tumor Markers and FDG-PETCT

For a long time, biochemical markers results were compared to those obtained by conventional morphological imaging modalities. In these circumstances, a high rate of false negatives was reported, and less than 20% of tumor marker elevations were associated with clinical and radiological findings. Consequently, these data have aroused doubts and criticisms in the scientific community about the value of tumor marker-guided follow-up also in case of BC [56]. During the last years, a growing number of scientific studies (Table 2) proved that whole-body FDG-PETCT scan is able to reduce the number of false negative cases by evidencing early tumor lesions previously undetectable by conventional morphological imaging exams. In this regard, it is important to consider two studies published in 2011 by Filippi et al. [58] and Evangelista et al. [59] who evidenced for the first time that hybrid FDG-PETCT scan was able to pick up cancer lesions, undetectable by conventional CT alone, in a cohort of about 40 asymptomatic BC patients with rising serum tumor markers. These observations were soon after corroborated in a third study by Champion et al. analyzing a large cohort of asymptomatic BC patients with rising CA15-3 and/or CEA tumor markers [60]. The ability of tumor markers to integrate PETCT exams for an optimal BC patient management during followup was also evidenced by Grassetto et al. who retrospectively studied 89 asymptomatic BC patients with rising CA15-3 levels and negative conventional imaging exams [61] and found that 40 out of 89 patients were positive at FDG-PETCT scan with tumor lesions detectable at level of chest wall, internal mammary nodes, lungs, liver, and skeleton. Moreover, in 23 out of 40 patients, a solitary lesion was detectable. Ultimately, in 2012, a study by Katayama et al. proved that change in tumor marker levels is primarily correlated with PET findings than CT, however, the hybrid pattern obtained by combining PET and CT imaging allow an optimal detection of FDG uptake to monitor disease progression, particularly in case of bone metastases, respect to other conventional imaging modalities [62].

## 5. Monitoring Response to Therapy in Advanced BC

Monitoring of therapy in patients with advanced BC is a critical issue in order to define cases responding to therapy from nonresponding ones [67]. Currently, the criteria of International Union against Cancer (UICC) are still used for assessing response to therapy, and they include physical examination, measurement of lesions, radiology, and isotope scanning [68]. Tumor markers levels measurement was not included in UICC criteria; however, two later multicenter studies showed that changes in serial concentrations of tumor markers, particularly CA15-3, correlate with response to therapy as well as with UICC criteria [69, 70]. In this regard, the actual guidelines from the European School of Oncology (ESO) suggest that “if tumor markers such as CA15-3 and CEA are elevated at time of treatment initiation, they can be helpful for therapy monitoring and long-term surveillance but they cannot be used solely for decision making with respect to change of therapy” [71]. Contrary to what is stated by ESO guidelines, the ASCO guidelines [52] do not suggest tumor marker measurement for monitoring response to therapy. However, since in about 10–20% of advanced BC the UICC criteria are not applicable (i.e., in patient with bone disease), the ASCO suggests tumor markers level measurement to have an early therapy response evaluation, but that tumor marker level alone is not sufficient for any therapy decision making.

## 6. Conclusions and Future Perspectives

The current routinely used serum tumor markers have limited usefulness for diagnosis and/or screening of BC due to their very low sensitivity and specificity as well as to the fact that they can be raised also in case of some benign conditions. For example, benign breast or ovarian disease and endometriosis may be associated with CA15-3 rising, while other conditions such as inflammatory bowel disease, pancreatitis, and gastritis may cause CEA increase [72]. Tumor markers level measurement at diagnosis may be only useful to point out those patients with advanced BC and then at risk to have liver involvement; however, it is not excluded that metastatic BC cases may present with normal serum concentrations.

As regards the usefulness of tumor markers for monitoring patients during followup, the debate is still open between scientific organizations (Table 1). In fact, the actual ASCO and ESMO guidelines do not suggest the use of tumor markers for monitoring BC patients during followup, and both confirm that they should be used only for advanced BC therapy monitoring, especially in cases where cancer lesions response, to therapy are not clinically evaluable. Conversely, the European Group for Tumor Markers (EGTM) [57] in agreement with the National Academy of Clinical Biochemistry (NACB) [57] sustains that serial evaluation of tumor markers levels is important for BC patient monitoring in order to get an early diagnosis of recurrence, since tumor markers rising often precede clinical or radiological signs of disease. Finally, the American College of Radiology (ACR) [54] and the European Association for Nuclear Medicine (EANM) [55] suggest that tumor markers increasing during followup may be an early warning able to highlight those patients needing molecular imaging investigations. In particular, according to recent studies (Table 2), both organizations reevaluated the role of tumor markers as an early warning able to highlight those patients at risk to have a recurrence due to clusters of tumor cells undetectable by conventional morphological imaging modalities. We believe that this last consideration is important since the biochemical markers results could integrate the diagnostic pathway for an early diagnosis of BC recurrence and, consequently, provide a better therapeutic intervention.

In our personal experience, CA15-3 proved to be a good serum tumor marker for those BC patients needing accurate molecular imaging investigations (PETCT) during followup. Our observations are in agreement with recent published studies suggesting that CA15-3 rising often precedes clinical or radiological signs of disease recurrence [61, 73]. Nevertheless, CA15-3 as well as other established biomarkers cited in this review does not fulfill the features of an ideal biomarker especially in terms of diagnostic sensitivity and specificity. On the basis of these diagnostics gaps, many research groups are conducting studies aimed at identifying new biomarkers able to diagnose BC at an early stage using minimally invasive approaches. In particular, during the last years, circulating noncoding molecules of RNA (miRNAs) are emerging as an innovative class of cancer biomarkers since found aberrantly expressed in different human cancers (tissues and serum) and featured by unprecedented levels of diagnostic specificity and sensitivity [74–77]. Despite this exciting discovery, common BC specific miRNAs have yet to emerge across studies, and it is too soon to interpret their functional role. In addition, comparing the circulating miRNAs profiling identified by different studies from different countries, only few of these miRNAs were corroborated by independent research groups [78]. On the basis of these pieces of evidence, it is essential to invest in larger study cohorts to validate a reproducible circulating-derived miRNAs signature to achieve true translational relevance and bring circulating miRNAs into routine diagnostics for early detection of BC, to predict outcome and in treatment planning. 

## Figures and Tables

**Table 1:** Current recommendations edited by international scientific organizations for the use of serum cancer biomarkers in clinical oncology.

Expert panel	Recommendation	Year of publication	Reference
ASCO	The use of CA15-3 and CEA is not recommended for routine surveillance of patients with breast cancer after primary therapy	2013	[52]

ESMO	Serum tumor markers (such as CA15-3 and/or CEA), if initially elevated, may be helpful in monitoring response, particularly in the case of nonmeasurable disease. However, a change in tumor markers alone should not be used as the only determinant for treatment decisions	2012	[53]

ACR	Localizing “occult” disease especially in the presence of clinical indicators such as elevated tumor markers	2012	[54]

EANM	Establishing and localizing disease sites as a cause for elevated serum markers (e.g., colorectal, thyroid, ovarian, cervix, melanoma, breast, and germ-cell tumours)	2010	[55]

NACB	CEA and CA15-3 are useful for therapy monitoring especially in patients with nonevaluable disease	2008	[56]

EGTM	CA15-3 and CEA are the most useful serum markers in patients with breast cancer. Serial determinations of these markers are useful in assessing prognosis, early detection of relapse (metastasis), and therapy monitoring	2005	[57]

**Table 2:** Studies proving the usefulness of performing PETCT scan on patients during followup with rising tumor markers for the detection of cancer lesions undetectable by conventional morphological imaging.

Study/year	Results	Remarks	Tumor markers	Reference
Filippi et al. Nucl Med Commun. 2011	FDG PETCT was positive in 36 out of 46 patients with rising biomarkers	The FDG-PET/CT scan plays an important role in restaging breast cancer patients with rising tumor markers and negative or equivocal findings in conventional imaging techniques	CEA and CA15-3	[58]

Evangelista et al. Eur J Nucl Med Mol Imaging. 2011	PETCT scan analysis was positive in 30 out of 40 patients with elevated tumor marker	FDG PETCT is more sensitive than CT for the evaluation of disease relapse; PETCT might be considered a complementary imaging technique during followup in patients with breast cancer	CA15-3	[59]

Champion et al. Cancer 2011	PETCT scans were positive in 181 patients (79.5%) and normal in 47 patients with rising CA15-3 and/or CEA	FDG PETCT imaging is an efficient technique to detect breast cancer recurrence suspected on tumor marker rising in asymptomatic patients	CEA and CA15-3	[60]

Grassetto et al. Eur J Radiol. 2011	Tumor deposits were detected in 40/89 patients by FDG PETCT	FDG PETCT may have a potential role in asymptomatic patients with rising markers and negative conventional imaging	CA15-3	[61]

Katayama et al. Ann Nucl Med. 2012	PETCT scan analysis was positive in 23 out of 47 patients with elevated tumor marker	The change in the tumor marker levels was substantially correlated with the PET findings and moderately correlated with the CT findings	CEA, I-CTP, CA15-3, BCA225, and NCC-ST-439	[62]
